# Comparison of the two surgery methods combined with accelerated rehabilitation in the treatment of lateral compression type 1 pelvic fractures in the elderly

**DOI:** 10.1186/s13018-023-04219-0

**Published:** 2023-09-28

**Authors:** Min Zou, Xin Duan, Mufan Li, Liangyu Ma, Miao Fang, Jiachen Sun

**Affiliations:** 1 Department of Orthopedics, Chengdu Second People’s Hospital, Chengdu, 610017 Sichuan People’s Republic of China; 2 Department of Orthopedics, No. 1 People’s Hospital of Chengdu, Chengdu, 610041 Sichuan People’s Republic of China; 3 Department of Orthopaedic Surgery, Zhongda Hospital, School of Medicine, Southeast University, Nanjing, 210009 People’s Republic of China

**Keywords:** INFIX, Cannulated screw, Older patients, Lateral compression type 1 pelvic fracture, Accelerated rehabilitation

## Abstract

**Background:**

Treating lateral compression type 1 (LC1) pelvic ring injuries in older patients is controversial. This study evaluated surgical treatments combined with ERAS for treating LC1 pelvic fractures in the elderly.

**Methods:**

In this retrospective study, patients who underwent surgery with INFIX (supra-acetabular spinal pedicle screws, and a subcutaneous connecting rod; the experimental group) or superior pubic ramus cannulated screw (the control group) fixation of LC1 pelvic fracture from January 2019 to January 2022 were reviewed. Injury radiography and computed tomography were performed to determine the Young–Burgess classification. All patients performed standardized early rehabilitation exercises after surgery and were followed up for > 12 months. After surgery, the Matta score and the visual analog scale (VAS) were evaluated, and the postoperative weight-bearing time and the length of stay (LOS) were recorded. The Barthel index and the Majeed score were evaluated at 4 months after surgery and at the last follow-up.

**Results:**

Fifty-three patients were included. Thirty-two patients included in the experimental group had a mean age of 75.0 ± 6.2 (range, 66–86) years, and the other 21 patients in the control group had a mean age of 74.6 ± 4.6 (range, 68–83) years. The mean follow-up time was 13.1 ± 1.6 (range, 12–18) months in the experimental group and 13.4 ± 1.3 (range, 12–16) months in the control group. There were no significant differences in follow-up time between the groups (*P* > 0.05). The mean VAS score, time to weight-bearing, and LOS were 2.0 ± 0.7 (range, 1–3), 1.1 ± 0.3 (range, 1–2) d, and 5.8 ± 0.9 (range, 4–7) d in the experimental group and 2.3 ± 1.2 (range, 1–5), 2.5 ± 1.6 (range, 1–7) d, and 6.1 ± 1.6 (range, 5–11) d in the control group, respectively. Between the two groups, there was a significant difference in the postoperative time to weight-bearing (*P* < 0.05), while there was no significant difference in the LOS (*P* > 0.05). No bedrest-related complications occurred in either group. The Matta score was 90.6% in the experimental group and 90.4% in the control group (*P* > 0.05). At the 4-months follow-up, the experimental group had a better Barthel index and Majeed score compared with the control group, which were 86.1 ± 6.2 (range, 70–95) vs. 81.2 ± 4.1 (range, 75–90) and 86.3 ± 3.3 (range, 78–91) vs. 80.3 ± 3.9 (range, 76–86), respectively. The experimental group had better early rehabilitation effect than the control group. There was no significant difference in Barthel index and Majeed score between the two groups at the last follow-up (*P* > 0.05).

**Conclusion:**

Both INFIX and intramedullary superior pubic ramus cannulated screws can successfully treat LC1 pelvic fractures and reduce bed rest complications among older patients.

## Introduction

Lateral compression pelvic type 1 (LC1) injury (according to the Young & Burgess classification [1]) is one of the most common pelvic injuries in older people. Known as fragility fractures of the pelvic ring (FFP), LC1 fragility fractures result from low-energy falls from a standing height or lower. They are more common among women, and most patients have osteoporosis. The incidence of these fractures increases with age, becoming a growing burden for the healthcare system [2, 3]. LC1 fractures include the fractures of rami pubis and/or sacrum. Rami fractures may be bilateral or unilateral, and sacral fractures may be complete or incomplete, minimal or un-displaced, and simple or comminuted [4]. Studies have shown that the anterior pelvic ring plays a role in preventing the collapse of the pelvic ring, and accounts for 40% of pelvic stability [5].

The treatment of LC1 stable pelvic fracture in the elderly has been controversial, with insufficient medical evidence to support which approach is the most effective [6, 7]. LC1 stable pelvic fractures in the elderly have traditionally been treated nonoperatively, especially for elderly patients with poor physical conditions [8, 9]. However, conservative treatment increased the patient's bed-rest complications such as pneumonia and venous thrombosis of lower limbs [7]. Therefore, surgical treatment is urgently needed for patients who cannot tolerate the pain caused by weight bearing [10].

In recent years, with the introduction of enhanced recovery after surgery (ERAS), surgical treatment has become less invasive. Intramedullary superior pubic ramus screws (often cannulated screws) and INFIX (supra-acetabular spinal pedicle screws and a subcutaneous connecting rod) are two such methods. Superior rami pubis fractures can be treated with percutaneous minimally invasive fixation by intramedullary cannulated screws with retrograde or antegrade placement [11]. Intramedullary cannulated screws have the advantages of requiring a small incision and short operation time as well as causing less bleeding, but they may easily damage blood vessels and nerves, need long radiation time [12], and have the risk of screw wrong placement and eventually the complication involving the femoral head, such as deepened femoral head vascularization fragility [13]. INFIX was originally developed to treat LC pelvic fractures in young patients [14]. It involves the percutaneous placement of screws in the pelvic bone and connects them with a bar under the skin. INFIX requires only a small incision and short operation time, leading to less bleeding and good fixation strength. However, it still has the disadvantages of causing discomfort and cutaneous nerve injury [15]. Traditional pelvic implants carry poor purchase in osteoporotic bone [16]. Biomechanical test results found that superior rami cannulated screws have superior stability compared to the standard surgical treatment and could provide fixed strength similar to superior ramus plating [17]. INFIX is a much more appealing surgical option for fragility fractures as a screw is fixed into the hard bone of the pelvis [18].

In addition, applying the ERAS philosophy in patients with pelvic fractures can improve the curative effect, reduce complications, improve the quality of life, and accelerate the functional rehabilitation of patients [19]. This study evaluated the early effects of different surgical treatments combined with ERAS in the treatment of LC1 pelvic ring injuries in elderly patients.

## Patients and methods

The Human and Ethics Committee approved this retrospective study for medical research at our hospital according to the Declaration of Helsinki. Written informed consent was obtained from all patients before inclusion in the study. We reviewed patients who underwent surgery with INFIX or canulated screws from January 2019 to January 2022. The patients treated with conservative treatment were excluded from this study due to the small number of cases and significantly more complications compared to the patients with surgery, and the detailed reasons are elaborated in the “Discussion” section. We employed the following inclusion criteria: (1) LC1 pelvic ring injuries, (2) age 65 years or older, (3) cannot tolerate pain, (4) being able to care for themselves before the injury. The exclusion criteria were as follows: (1) pathological fracture; (2) fractures of other parts of the lower limbs; (3) unable to move autonomously before the injury; and (4) cognitive dysfunction such as Alzheimer’s disease. All patients were classified into the experimental group with INFIX surgery or the control group with cannulated screw infixation. Uniform measures to accelerate recovery were initiated upon admission. Demographic data, injury characteristics, and surgery-related data were extracted from medical records. Injury radiography and CT were performed to determine the Young–Burgess classification. Analgesics were applied according to the patient’s condition to relieve pain. Surgery was performed within 1–2 days after admission. Patients treated conservatively were given bed immobilization for 1 week, and then the time of weight-bearing was determined according to the pain, and bed rest was the main treatment within 3 weeks after injury.

### Surgical procedure

#### INFIX

**Fig. 1:**
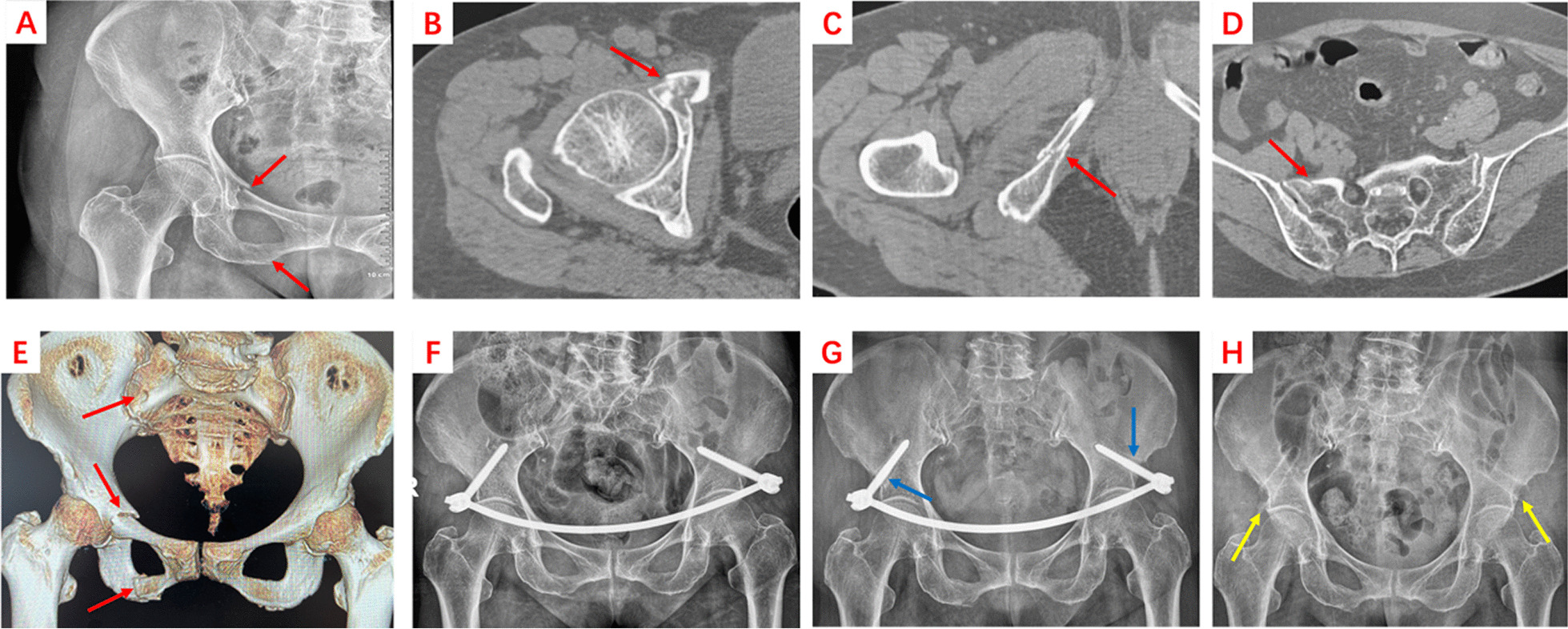
An 86-year-old patient with LC1 pelvic fracture underwent surgery with INFIX. **A** X-ray examination on admission showed fractures of the upper and lower rami of the right pubic bone (red arrows). **B**–**E** CT scan and three-dimensional reconstruction indicated incomplete fractures of the right superior and inferior ramus of the pubis and ipsilateral sacrum (red arrow). **F** X-ray examination taken after INFIX fixation. **G** At 4 months after surgery, the X-ray image showed that the fracture had healed and there was an empty shadow around the screw, indicating that the screw was loose (blue arrow). And INFIX fixation was subsequently removed. **H** At 13 months after surgery, the X-ray image showed a small amount of heterotopic ossification was seen in the anterior inferior iliac spine (yellow arrow)

After administering anesthesia, oblique incisions about 2–3 cm long were made on both sides of the anterior inferior iliac spine. The deep fascia and muscles were bluntly separated along the incision to fully expose the anterior inferior iliac spine. The needle insertion point, located above the acetabular, pointing to the posterior superior iliac spine and between the internal and external plates of the iliac bone, was confirmed by X-ray. Pedicle screws with a diameter of 7.5 mm and a length of 75–85 mm were inserted on both sides after the pedicle mouth opener was opened, and the screws protruded from the bone surface 15–30 mm (depending on the individual patient). A subcutaneous tunnel was established in the fold area on both sides of the abdomen and groin from the bilateral iliac spine incision with a long vascular clamp. A connecting rod with a diameter of 6 mm and an appropriate length was passed through the subcutaneous tunnel and inserted into the tail of each screw. The screw tail cap was stretched and reduced according to the compression of the fracture (Fig. 1).

### Retrograde cannulated screw

**Fig. 2:**
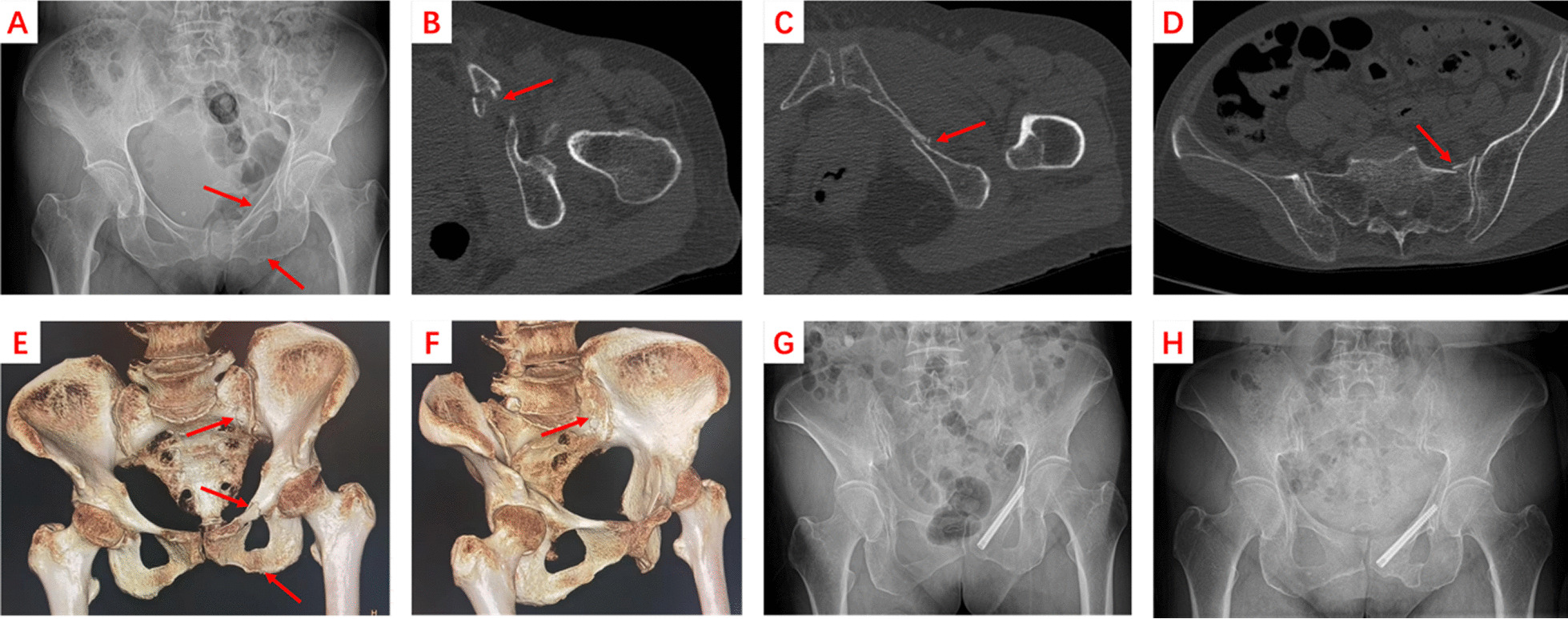
A 76-year-old patient with LC1 pelvic fracture underwent superior ramus cannulated screw surgery. **A** An X-ray examination on admission showed a fracture of the left superior and inferior ramus of the pubic bone (red arrow). **B**–**F** CT scan and three-dimensional reconstruction indicated incomplete fracture of the right superior and inferior ramus of pubic ramus and ipsilateral sacrum (red arrow). **G** X-ray image taken after the operation with a superior ramus cannulated screw. **H** At 16 months after surgery, the X-ray image showed that the fracture had healed and there was no loose or displaced internal fixation. The patient had no discomfort, so the screw was not removed

The canulated screws were placed using the retrograde method. The entry point was located below the pubic tubercle. A 2.0-mm Kirschner wire was used as a needle guide. After confirming the entry point under fluoroscopy, the angle was adjusted and maintained, and the Kirschner wire was slowly drilled into the intramedullary of the superior pubic ramus and passed through the fracture. The fluoroscopy of the pelvic inlet and obturator outlet was used to confirm that the Kirschner wire had passed through the fracture and had been located in the superior pubic ramus. Finally, a 7.0 mm full-thread cannulated screw was inserted through the needle to fix the fracture. (Fig. 2).

### Accelerated rehabilitation protocol

After admission, patients were given bed immobilization and non-steroidal anti-inflammatory analgesia if necessary. A gastric mucosa protectant was used to prevent stress ulcers. Oral education, a multimedia video animation, and a demonstration were used to conduct preoperative education to treat anemia and hypoproteinemia. A comprehensive assessment was made of the activity level before the injury and the results of an electrocardiogram. A heart color Doppler ultrasound, a test of lung function, and other related tests were performed to determine whether the patient was likely to tolerate the risks of surgery and anesthesia and achieve active control of blood pressure and blood glucose. The target blood glucose control in diabetic patients is about 10 mmol/L. After the contraindications such as active bleeding and injury were excluded, a subcutaneous injection of low-molecular-weight heparin (Sanofi, France) was started 6 h after acute injury for anticoagulation therapy combined with a lower extremity pressure pump to encourage patients to actively move their lower extremities, and D-dimer changes were dynamically detected. There was no need for a routine enema before surgery. First-generation cephalosporin antibiotics were given once at 30 min to 1 h before surgery and once after surgery.

Regarding preoperative diet management, the usual fasting time was 8 h, and drinking was forbidden for 4–6 h. Lumbar anesthesia was preferred if the patient could tolerate pain while lying on their side and there were no contraindications; otherwise, general anesthesia was used. After eating 4 h after surgery, the patients were encouraged to sit up and move in bed; 24 h after surgery, the patients should stand and walk with their weight on the ground with the help of a rehabilitation physician. If the patient could not tolerate the pain, the time to weight-bearing on the ground was postponed to ensure the patient’s safety. We started anti-osteoporosis therapy with a subcutaneous injection of calcitonin immediately after surgery.

### Postoperative assessment

The visual analog scale (VAS) was used to assess pain perception after the operation. The Matta score was used to evaluate the fracture reduction and record the time to weight-bearing after surgery and the length of stay (LOS). All patients were followed up at 1, 2, and 4 months and every 6 months after surgery, and complications were recorded. The Barthel index and Majeed score were used to evaluate the patients at 4 months after surgery and at the last follow-up to assess activities of daily living and functional recovery.

### Statistical analysis

Statistical analyses were conducted with SPSS version 16.0 (SPSS Inc., Chicago, IL, USA). All measurement data were tested for whether they followed a normal distribution using the Kolmogorov–Smirnov Z test. Comparisons of variables between the baseline and the endpoint were analyzed using paired t-tests when the distribution was normal; otherwise, the Wilcoxon signed-rank test was used. A *P* value of < 0.05 was defined as significant.

## Results

Fifty-nine elderly patients with LC1 injuries received surgical treatment in this study. Among them, 53 cases were followed up for more than 12 months, and 6 cases were lost to follow-up (outpatients lost to follow-up or telephone errors). The 53 patients included 32 cases in the experimental group and 21 cases in the control group. The patient characteristics are presented in Table 1. The mean follow-up time was 13.1 ± 1.6 (range, 12–18) months in the experimental group and 13.4 ± 1.3 (range, 12–16) months in the control group. Of the 53 patients, 12 were male and 20 were female in the experimental group, with a mean age of 75.0 ± 6.2 (range, 66–86) years; nine were male and 12 were female in the control group, with a mean age of 74.6 ± 4.6 (range, 68–83) years. There were 16 and 10 patients with one or more diseases (e.g., hypertension, diabetes, coronary heart disease, chronic obstructive pulmonary) in the experimental group and in the control group, respectively. Three patients had deep vein thrombosis in the experimental group. The initial injuries in the experimental group were caused by falls in 23 cases and traffic accidents in 9 cases, while those in the control group were falls in 16 cases and traffic accidents in 5 cases. Pelvic fractures included 27 unilateral vs. 5 bilateral rami fractures in the experimental group and 18 unilateral vs. 3 bilateral rami fractures in the control group. Four cases of sacral fractures were complete in the experimental group and two cases in the control group. The mean time from injury to surgery was 3.2 ± 1.7 (range, 2–9) d in the experimental group and 3.0 ± 1.1 (range, 2–6) d in the control group. The patient characteristics between the two groups were not significantly different (*P* > 0.05).

**Table 1:** Patient characteristics

Characteristics	Infixation (*n* = 53)		*P* value
	INFIX (*n* = 32)	Canulated screw (*n* = 21)	
Age, years, mean ± SD	75 ± 6.2	74.6 ± 4.6	0.970
Sex, male/female	12/20	9/12	0.682
Pelvic fracture			
Rami fractures, unilateral/bilateral	27/5	18/3	0.561
Sacral fractures, complete/incomplete	4/28	2/19	0.632
^a^Basic diseases	16	10	0.561
^b^Associated injuries	6	3	0.494
Mechanism of injury, fall/traffic accident	23/9	16/5	0.634
Time to surgery, days, mean ± SD	3.2 ± 1.7	3.0 ± 1.1	0.627

^a^Including one or more of the hypertension, diabetes, coronary heart disease, lung infection

^b^Including one or more of the Scalp injury, rib fracture, DVT

The clinical data and postoperative assessment are presented in Table 2. The mean operative time was 31.3 ± 3.9 (range, 25–40) min in the experimental group and 15.0 ± 4.1 (range, 10–22) min in the control group. The mean intraoperative blood loss was 3.4 ± 1.3 (range, 2–5) mL in the experimental group and 7.9 ± 14 (range, 2–50) mL in the control group. The number of intraoperative fluoroscopies was 32.6 ± 7.7 times (20–55 times) in the experimental group and 21.2 ± 3.9 times (15–30 times) in the control group. The operative time, intraoperative blood loss, and radiation times between the two groups were significantly different (*P* < 0.05). The mean VAS score on the first postoperative day was 2.0 ± 0.7 (range, 1–3) in the experimental group and 2.3 ± 1.2 (range, 1–5) in the control group. Pain was significantly reduced in both groups after surgery (*P* < 0.05), but the mean VAS score had no significant difference between the two groups (*P* > 0.05). For the Matta score, the “excellent and good” experimental group rate was 90.6% and that of the control group was 90.4%. Postoperative weight-bearing time averaged 1.1 ± 0.3 (range, 1–2) d in the experimental group and 2.5 ± 1.6 (range, 1–7) d in the control group, which was significantly different (*P* < 0.05). The mean hospital stay was 5.8 ± 0.9 (range, 4–7) d in the experimental group and 6.1 ± 1.6 (range, 5–11) d in the control group (*P* > 0.05).

**Table 2:** Clinical data and postoperative assessment

Results	Infixation (*n* = 53)		*P* value
	INFIX (*n* = 32)	Canulated screw (*n* = 21)	
Operative time, minutes, mean ± SD	31.3 ± 3.9	15.0 ± 4.1	0.001
Intraoperative blood loss, mL, mean ± SD	3.4 ± 1.3	7.9 ± 14	0.073
intraoperative fluoroscopy, times, mean ± SD	32.6 ± 7.7	21.2 ± 3.9	0.001
Visual analogue scale score, mean ± SD	2.0 ± 0.7	2.3 ± 1.2	0.176
Time to weightbearing, mean ± SD	1.1 ± 0.3	2.6 ± 1.6	0.001
LOS, mean ± SD	5.8 ± 0.9	6.1 ± 1.6	0.350
Follow-up time, months, mean ± SD	13.1 ± 1.6	13.4 ± 1.3	0.627
Fracture healing time, weeks, mean ± SD	7.2 ± 0.9	7.3 ± 1.0	0.531
Complications			
Lateral femoral cutaneous nerve injury	2	0	–
Vessel injury	0	2	–
Ineffective internal fixation	0	2	–
Heterotopic ossification	8	0	–
Internal fixation loosening	14	6	–
Matta score, excellent-effective rate (%)	90.6	90.4	–
Barthel index, 4/last, month, mean ± SD	86.1 ± 6.2 94.2 ± 5.2	81.2 ± 4.1 93.1 ± 4.6	0.003 0.428
Majeed score, 4/last, month, mean ± SD	86.3 ± 3.3 96.1 ± 0.8	80.3 ± 3.9 95.8 ± 0.7	0.001 0.391

In the experimental group, two patients had lateral femoral cutaneous nerve injury, which had recovered 3 months later with nutritional nerve treatment. At the last follow-up, 14 patients had internal fixation loosening and fixation were subsequently removed; eight patients had grade I heterotopic ossification, which did not affect movement, or cause pain and discomfort, so no treatment were given; one patient had sartorius muscle compression, which was relieved after symptomatic treatment, and the internal fixation was removed after the fracture healed; and one patient had postoperative DVT aggravation. Calf muscle vein thrombosis developed in the femoral vein and posterior tibial vein, and an inferior vena cava filter was placed. In the control group, there were two cases of vascular injury and bleeding was stopped by compression, six cases had internal fixation loosening, and two cases had internal fixation failure in which the screw penetrated the bone, and those fixations were removed.

Fracture healing was achieved in all patients. The average fracture healing time was 7.1 (range, 6–9) weeks in the experimental group and 7.3 (range, 6–10) weeks in the control group, with no significant difference (*P* > 0.05). Four months after the operation, the Barthel index and Majeed score were 86 (range, 70–95) and 86 (range, 78–91) in the experimental group and 81 (range, 75–90) and 80 (range, 76–86) in the control group, respectively. At the last follow-up, the Barthel index and Majeed score were 94 (range, 85–100) and 96 (range, 95–98) and 93 (range, 85–100), and 95 (range, 95–97) in the experimental and control groups, respectively. There was a significant difference between the two groups in the Barthel and Majeed scores after 4 months (*P* < 0.05), but there was no significant difference at the last follow-up (*P* > 0.05). No patient had wound dehiscence, deep or superficial infection, stiffness, and none of the patients developed bed-related complications such as pneumonia and bedsores.

## Discussion

The results of this study confirm that INFIX and super ramus cannulated screws are effective methods for the minimally invasive treatment of LC1 pelvic fractures in older patients. Combined with the ERAS concept, both can relieve pain, enable early weight-bearing walking, result in good function, restore daily living ability, and reduce complications related to bed rest.

Conservative treatment of LC1-type pelvic injury in the elderly remains a priority [6, 8, 20]. However, this is controversial. Tucker et al. [21] found that older patients with minimally displaced unstable LC1 injuries had similar hospital courses as those with intertrochanteric femur fractures. These patients are advised to undergo internal fixation or joint replacement within 36 h of injury to reduce the incidence of bed-related complications. Van et al. [22] reported that 99 elderly patients with pelvic pubic rami fractures were treated conservatively. The incidence of complications during hospitalization was 20.2%, and the 1-year mortality rate was 24.7% and the 5-years mortality was 64.4%, 33% of the patients were unable to walk autonomously at the last follow-up. The inability of such patients to tolerate pain leads to increased bedtime and increased need for care, while reducing the confidence and mood of elderly patients, seriously affecting their quality of life [23]. In a long-term follow-up study of patients with low-energy pelvic injuries, Kugelman et al. [24] found that 26% of patients lost the ability to walk independently and required an assistive walking device after nonsurgical treatment. Some scholars use lateral stress radiographs or examinations under anesthesia to determine whether there is occult pelvic instability and decide which treatment to use [25, 26]. Some judge the stability of the fracture by its morphological characteristics. Oblique fractures of the pubic ramus, comminuted fractures, and sacral zone I/II fractures have recessive instability and easily shift to later-stage fractures, suggesting that surgical treatment is warranted for these fractures [27]. Moreover, treatment is also determined by CT classification. Patients with type II group fragility fractures who cannot tolerate painful activity within 3–5 days are indicated for surgery [10].

Currently, the key points of treatment of LC1 pelvic injuries in the elderly are pain relief, early weight-bearing walking, reducing complications of bed rest, and early recovery of activities of daily living [21, 28]. Therefore, for all elderly patients with type LC1 pelvic fracture who come to our hospital, we use pain as the reference standard for treatment [29]. If the patient can walk 15 feet with a painful weight, they will be treated conservatively. Otherwise, they will be treated surgically. When we reviewed the patients with type LC1 pelvic fracture, 6 of them were treated with conservative treatment. During the rehabilitation process, 2 patients had pulmonary infection and 1 patient had lower extremity venous thrombosis. After 4 months, the average Barthel score was 73.3 ± 4.1, and the average Majeed score was 75.2 ± 3.0. These patients had a long recovery time and more complications, which did not meet the current requirements of enhanced recovery in elderly patients, and the number of cases was small, so they were not included in the study. For the patients undergoing surgery, especially those without complete sacral fracture, anterior ring fixation is recommended, as posterior ring fixation alone is insufficient to provide stability [27]. In this study, most sacrum fractures were incomplete, and even if it was complete, there was almost no displacement, so the posterior ring was stable. Therefore, we only fixed the anterior ring.

Jennifer et al. reported that patients with LC1 fractures who underwent surgery were ambulatory 1.7 d earlier than those treated conservatively [30]. In the control group, two patients had delayed weight-bearing time due to local hematoma formation and the aggravation of pain caused by intraoperative vascular injury. In these two patients, the internal fixation penetrated the cortex due to the wrong intraoperative fluoroscopy angle, which delayed the weight-bearing time. There was a significant difference in the VAS score in both groups before and after surgery. After surgery, there were no significant differences in pain perception between the groups, indicating that the two surgical treatments can stabilize fractures and relieve pain. Additionally, there were significant differences in operation time and intraoperative fluoroscopy times between these two groups. The control group had smaller incisions, underwent a simpler operation, and had a shorter operation time. Besides, two patients in the control group had vascular injury complications, which led to increased blood loss, but there was no significant difference between these two groups in intraoperative blood loss. Because of the small displacement of this type of fracture, the postoperative Matta score was > 90% in both groups. At the 4-months follow-up after the operation, the Barthel score of the experimental group was higher than that of the control group (*P* < 0.05), but it was not statistically significant at the last follow-up. In terms of the Majeed pelvic function score, the experimental group had a significantly better score than the control group at 4 months post-surgery but there was no significant difference in the scores at the last follow-up, which indicated that patients given the INFIX surgery recovered faster in the early postoperative period.

In a retrospective study of 913 patients with pelvic ring injuries, Ochenjele et al. [31] found that 6% of surgical fixation failed and required revision surgery. INFIX could cause lateral femoral cutaneous nerve injury, hip joint capsule injury, and muscle compression [32]. In this study, two patients in the experimental group had unilateral lateral femoral cutaneous nerve injury. This may have been due to a traction injury of the nerve caused by the blind separation of soft tissue and making a small incision at the beginning of the implementation of the surgical plan. Another patient had sartorius muscle compression, which manifested as pain in the groin in the sitting position. This might be due to the screw being placed too deep in the bone, resulting in a small gap between the screw and the connecting rod. Additionally, one patient in the experimental group had aggravated venous thrombosis of the lower extremities, after investigating the medical history, we found that the patient had used coagulation drugs for gastrointestinal bleeding about 1 month, which was considered to be the cause of thrombosis aggravation. Moreover, older patients are more likely to have osteoporosis and are prone to experience internal fixation failure and loosening during fracture healing [33]. The screw loosened in 14 patients in the experimental group and in six patients in the control group. However, both methods can provide a stable fixation effect for early fracture healing in older patients before fixation loosening. The INFIX is usually removed within 4–6 months after surgery if the fracture has healed, whereas a canulated screw internal fixation is not removed if there is no discomfort.

Enhanced recovery of elderly patients includes special attention to pain and osteoporosis to obtain the maximum benefit at the minimum cost and avoid secondary injury [34]. In the past, the importance of ERAS was not realized. Due to osteoporosis in elderly patients and the fear of failure of internal fixation, even patients undergoing surgery were afraid to exercise in the early stage. However, INFIX and canulated screws can provide good fixation for elderly patients with osteoporosis. In this study, both groups had no pulmonary infections, urinary tract infections, bedsores, additional falls, or other complications, which reflects not only the benefits of surgery to patients, but also the joint effect of enhanced recovery after the ERAS measures.

There are also some limitations of this study. First, this is a retrospective analysis, and the sample size is small. Second, the follow-up period of this study was short. Finally, although both INFIX and cannulated screws can stabilize pelvic fractures in the early stage, there are certain complications, such as fixation loosening in the late stage. At present, we believe that these two surgical methods are already the preferred options for treating LC1 pelvic fractures, and there is no more effective surgical treatment with fewer complications.

## Conclusion

For older patients with LC1 fragility pelvic injury, if they cannot tolerate pain and weight-bearing, surgical treatment is recommended. In this study, we found that INFIX and superior pubic ramus cannulated screws are effective surgical methods to treat LC1 pelvic fractures in older patients. Combined with ERAS measures, patients after surgery can achieve satisfactory function and daily life ability earlier.

## Data Availability

Yes.
